# Lobectomy versus sublobar resection on survival in patients with stage T1-2N0M0 small cell lung cancer

**DOI:** 10.1371/journal.pone.0324315

**Published:** 2025-05-14

**Authors:** Xue Yin, Huan-Wei Liang, Yang Liu, Wei Huang, Xin-Bin Pan

**Affiliations:** 1 Department of Oncology, The Central Hospital of Wuhan, Tongji Medical College, Huazhong University of Science and Technology, Wuhan, Hubei, P.R. China; 2 Department of Radiation Oncology, Guangxi Medical University Cancer Hospital, Nanning, Guangxi, P.R. China; European Institute of Oncology: Istituto Europeo di Oncologia, ITALY

## Abstract

**Purpose:**

To compare survival outcomes of lobectomy versus sublobar resection in patients diagnosed with stage T1-2N0M0 small cell lung cancer (SCLC).

**Materials and methods:**

A comprehensive retrospective analysis was conducted using data from the Surveillance, Epidemiology, and End Results database. The Kaplan-Meier method was utilized to estimate cancer-specific survival (CSS) and overall survival (OS) between stage T1-2N0M0 SCLC patients undergoing sublobar resection and those receiving lobectomy.

**Results:**

A total of 185 patients were included in the analysis, with 64 undergoing sublobar resection and 121 receiving lobectomy. Before propensity score matching, lobectomy was associated with significantly better CSS (hazard ratio [HR] =, 95% confidence interval [CI]: 0.32, P = 0.006) and OS (HR =, 95% CI: 0.39, P = 0.005) compared to sublobar resection. Multivariable Cox regression analysis confirmed that lobectomy was an independent predictor of improved CSS (HR =, 95% CI: 0.24, P = 0.003) and OS (HR =, 95% CI: 0.36, P = 0.013). After propensity score matching, the differences in CSS (HR =, 95% CI: 0.36, P = 0.177) and OS (HR =, 95% CI: 0.46, P = 0.234) between the two surgical approaches were not statistically significant,

**Conclusion:**

Lobectomy may offer improved survival outcomes compared to sublobar resection in patients with stage T1-2N0M0 SCLC.

## Introduction

Small cell lung cancer (SCLC), an exceptionally aggressive neuroendocrine malignancy accounting for approximately 15% of pulmonary neoplasms, continues to present formidable clinical challenges due to its rapid progression and propensity for early metastasis [1]. Although traditionally diagnosed at advanced stages (extensive disease in 70% of cases), evolving lung cancer screening paradigms utilizing low-dose computed tomography have enhanced early detection rates, particularly identifying localized tumors amenable to curative-intent therapies [2].

For stage T1-2N0M0 SCLC patients, the current therapeutic cornerstone of concurrent chemoradiotherapy achieves modest 5-year overall survival (OS) rates of 25–30% [3,4]. Emerging evidence from retrospective analyses challenges this paradigm, suggesting surgical resection may confer superior oncologic outcomes in carefully selected cases with preoperative histopathological confirmation [5–8]. Notably, lobectomy, the anatomical resection standard for non-small cell lung cancer (NSCLC), demonstrates particularly promising results, with observational studies reporting 5-year OS approaching 50% in early-stage SCLC cohorts [9,10].

This survival dichotomy raises critical questions regarding optimal surgical strategy. While sublobar resection (encompassing wedge resection and segmentectomy) theoretically preserves pulmonary parenchyma and may benefit functionally compromised patients, its oncologic equivalence to lobectomy remains unproven in the unique biological context of SCLC. Our study addresses this knowledge gap through comparative effectiveness analysis of lobar versus sublobar resection in stage T1-2N0M0 SCLC, utilizing population-level data to inform surgical decision-making.

## Materials and methods

We conducted a retrospective cohort analysis using the National Cancer Institute’s Surveillance, Epidemiology, and End Results (SEER) database (2000–2020). Pre-treatment evaluations, including histopathological confirmation, imaging staging, and biomarker assessment, were systematically implemented as per guidelines [11].

Histopathological verification was achieved via computed tomography-guided core needle biopsy for peripheral lesions or endobronchial ultrasound-guided transbronchial needle aspiration for central lesions or suspected mediastinal involvement. Imaging staging comprised contrast-enhanced thoracic computed tomography to evaluate primary tumor extent and nodal involvement. Brain magnetic resonance imaging, bone scans and the 18-fluorodeoxyglucose positron emission tomography/computed tomography were performed for distant metastasis detection. Serum neuron-specific enolase and pro-gastrin-releasing peptide were quantified at initial diagnosis and at 3-month intervals during follow-up. Biomarker elevation informed differential diagnosis and prompted metastatic workup but did not preclude eligibility.

The study cohort comprised adults (≥18 years) with pathologically confirmed pT1-2N0M0 SCLC (AJCC 7th edition staging) who underwent curative-intent surgical resection. Inclusion required complete survival data and exclusion criteria encompassed: [1] presence of multiple primary malignancies [2], incomplete staging evaluation [3], receipt of neoadjuvant therapy, and [4] cases managed without curative-intent surgical resection.

Demographic variables encompassed age, sex, and race. Tumor characteristics included primary site, laterality, histological grade, and T-stage stratification. Treatment parameters recorded radiation therapy administration and chemotherapy receipt. The primary exposure variable was surgical approach: lobectomy versus sublobar resection. Additionally, we gathered follow-up data to assess survival outcomes, including cancer-specific survival (CSS) and OS.

Categorical variables were compared using χ² tests or Fisher’s exact tests. Survival distributions were estimated via Kaplan-Meier methodology with between-group comparisons using log-rank tests. Multivariable Cox proportional hazards regression analyses were performed to identify independent prognostic factors, adjusting for clinically relevant confounders including age, sex, race, tumor characteristics, and adjuvant therapies.

To address selection bias inherent in surgical cohort studies, we implemented propensity score matching (PSM) using a non-parsimonious logistic regression model incorporating all baseline variables. A 1:1 nearest-neighbor matching algorithm with caliper width of 0.1 created balanced comparison groups. Post-matching balance was assessed through standardized mean differences (< 0.1 considered acceptable).

All analyses were performed using SPSS 26.0 (IBM Corp.) and R 4.2.2 (R Foundation), with two-tailed P < 0.05 defining statistical significance.

## Results

### Patient selection

**Fig 1:**
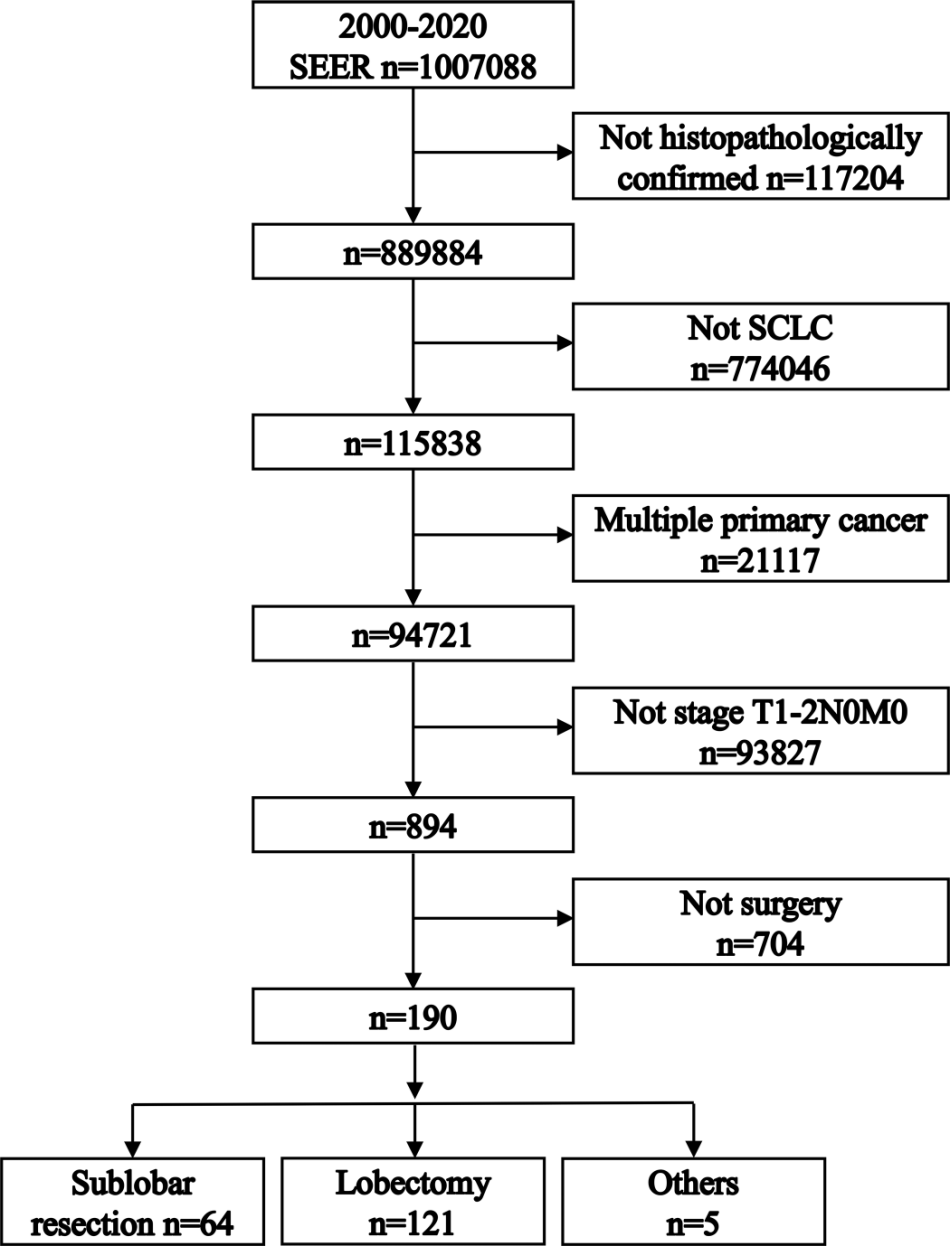
Flowchart depicting the patient selection process for the study. This diagram illustrates the sequential steps taken to identify and include the final cohort of 185 patients with stage T1-2N0M0 small cell lung cancer (SCLC) who underwent either sublobar resection or lobectomy, from an initial pool of 1,007,088 lung cancer patients.

The SEER database interrogation initially identified 1,007,088 lung cancer diagnoses (2000–2020). Sequential application of inclusion/exclusion criteria yielded 190 T1-2N0M0 SCLC cases. After excluding 5 patients with incomplete records, the final analytical cohort comprised 185 surgically treated patients. Of these, 64 patients received sublobar resection, while 121 underwent lobectomy. Fig 1 details the selection flowchart.

### Baseline characteristics

Table 1 presents pre-PSM and post-PSM demographic comparisons. The baseline characteristics of patients in both the sublobar resection and lobectomy groups are summarized in Table 1. Prior to PSM, there were significant differences in treatment modalities between the two groups (P = 0.009). However, following PSM, the comparison became balanced across all key variables, including age, sex, race, tumor location, tumor laterality, histological grade, T stage, and treatment modality. This adjustment ensured that any observed differences in survival outcomes could be more reliably attributed to the type of surgery performed rather than confounding variables.

**Table 1:** Patient characteristics.

	The unmatched cohort		P	The PSM cohort		P
	Sublobar resection (n = 64)	Lobectomy (n = 121)		Sublobar resection (n = 50)	Lobectomy (n = 50)	
Age			0.999			0.999
<67	26 (40.6%)	66 (54.5%)		22 (44.0%)	22 (44.0%)	
≥67	38 (59.4%)	55 (45.5%)		28 (56.0%)	28 (56.0%)	
Sex			0.999			0.999
female	37 (57.8%)	70 (57.9%)		28 (56.0%)	28 (56.0%)	
male	27 (42.2%)	51 (42.1%)		22 (44.0%)	22 (44.0%)	
Race			0.825			0.999
white	61 (95.3%)	117 (96.8%)		49 (98.0%)	49 (98.0%)	
black	1 (1.6%)	2 (1.6%)		1 (2.0%)	1 (2.0%)	
others	2 (3.1%)	2 (1.6%)				
Site			0.060			0.999
upper lobe	48 (75.0%)	68 (56.2%)		37 (74.0%)	37 (74.0%)	
middle lobe	4 (6.2%)	8 (6.6%)		3 (6.0%)	3 (6.0%)	
lower lobe	11 (17.2%)	41 (33.9%)		9 (18.0%)	10 (20.0%)	
others	1 (1.6%)	4 (3.3%)		1 (2.0%)	0 (0.0%)	
Laterality			0.516			0.311
left	32 (50.0%)	53 (43.8%)		24 (48.0%)	18 (36.0%)	
right	32 (50.0%)	68 (56.2%)		26 (52.0%)	32 (64.0%)	
Grade			0.107			1.000
III/IV	46 (71.9%)	71 (58.7%)		17 (34.0%)	17 (34.0%)	
I/II/unknown	18 (28.1%)	50 (41.3%)		33 (66.0%)	33 (66.0%)	
T stage			0.106			0.402
T1N0M0	47 (73.4%)	73 (60.3%)		35 (70.0%)	30 (60.0%)	
T2N0M0	17 (26.6%)	48 (39.7%)		15 (30.0%)	20 (40.0%)	
Treatment			0.009			0.765
surgery alone	20 (31.2%)	42 (34.7%)		17 (34.0%)	20 (40.0%)	
surgery+chemotherapy	16 (25.0%)	52 (43.0%)		16 (32.0%)	12 (24.0%)	
surgery+radiotherapy	1 (1.6%)	2 (1.6%)		1 (2.0%)	2 (4.0%)	
surgery+chemoradiotherapy	27 (42.2%)	25 (20.7%)		16 (32.0%)	16 (32.0%)	

PSM: propensity score matching.

### Logistic regression analysis for surgical approach selection

**Fig 2:**
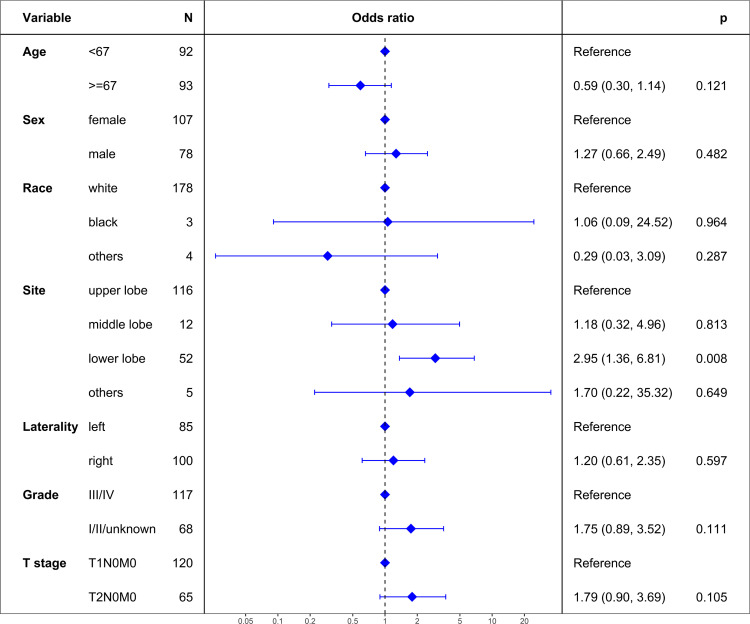
Results from logistic regression analysis identifying factors influencing the surgical approach selection in the study population. The analysis highlights significant variables that favor the selection of lobectomy over sublobar resection, with a specific focus on the lower lobe location as a significant determinant.

Logistic regression analysis was performed to identify factors associated with the selection of lobectomy over sublobar resection. The analysis revealed that the tumor’s lower lobe location significantly favored lobectomy (adjusted odds ratio = 2.95, 95% confidence interval [CI]: 1.36–6.81; P = 0.008). No other variables, including age, sex, race, tumor laterality, grade, or T stage, were found to have a statistically significant association with the choice of surgical approach (Fig 2).

### Cancer-specific survival

**Fig 3:**
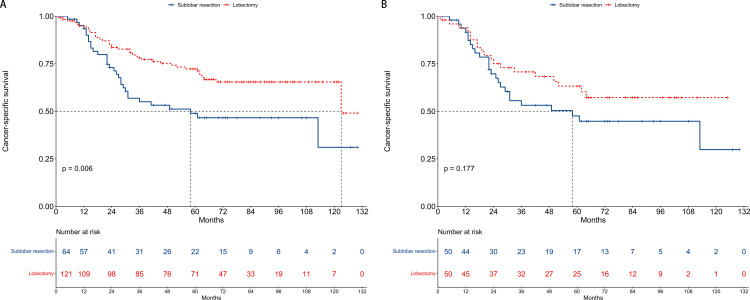
Kaplan-Meier curves for cancer-specific survival comparing sublobar resection and lobectomy groups. (A) The unmatched cohort: This panel shows the cancer-specific survival rates for patients who underwent sublobar resection versus those who had lobectomy before propensity score matching, indicating a significant survival advantage for lobectomy. (B) The propensity score matching cohort: This panel depicts the cancer-specific survival rates after matching, demonstrating no statistically significant difference between the two surgical approaches.

**Fig 4:**
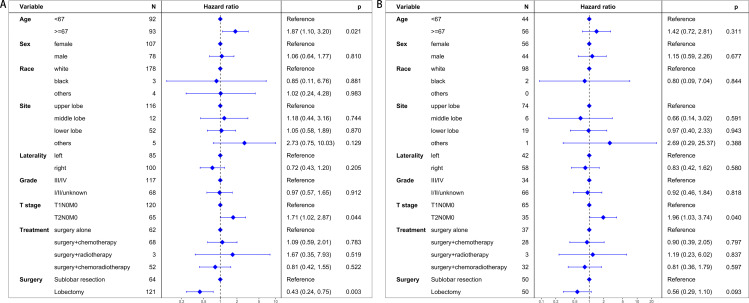
Multivariate regression analysis evaluating prognostic factors for cancer-specific survival. (A) The unmatched cohort: This analysis identifies lobectomy as an independent predictor of improved cancer-specific survival in the unmatched patient population. (B) The propensity score matching cohort: This panel shows the adjusted analysis post-matching, indicating that the initial survival advantage of lobectomy is not statistically significant after controlling for patient characteristics.

In the pre-PSM cohort, the median CSS was 58 months for patients undergoing sublobar resection and 123 months for those receiving lobectomy. Univariate analysis demonstrated a significant survival advantage for patients in the lobectomy group (hazard ratio [HR] = 0.51, 95% CI: 0.32–0.82, P = 0.006), as illustrated in Fig 3A. Multivariate analysis confirmed that lobectomy was an independent predictor of improved CSS (HR = 0.43, 95% CI: 0.24–0.75, P = 0.003), reinforcing its potential as a superior surgical option (Fig 4A).

Following PSM, the median CSS remained 58 months in the sublobar resection group, while the median CSS for the lobectomy group was not reached, indicating prolonged survival. However, the survival benefit of lobectomy was not statistically significant after matching (HR = 0.66, 95% CI: 0.36–1.21, P = 0.177), as shown in Fig 3B. Multivariate analysis echoed these findings, with no significant difference in CSS between the two groups (HR = 0.56, 95% CI: 0.29–1.10, P = 0.093, Fig 4B).

### Overall survival

**Fig 5:**
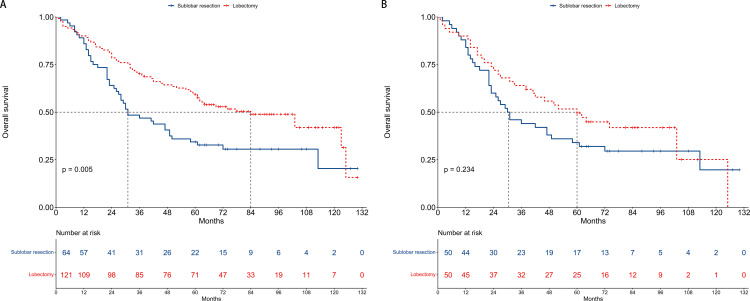
Kaplan-Meier curves for overall survival comparing sublobar resection and lobectomy groups. (A) The unmatched cohort: This panel illustrates the overall survival rates for the two surgical approaches before propensity score matching, with lobectomy showing a significant survival benefit. (B) The propensity score matching cohort: This panel presents the overall survival rates post-matching, showing no significant difference in survival between sublobar resection and lobectomy.

**Fig 6:**
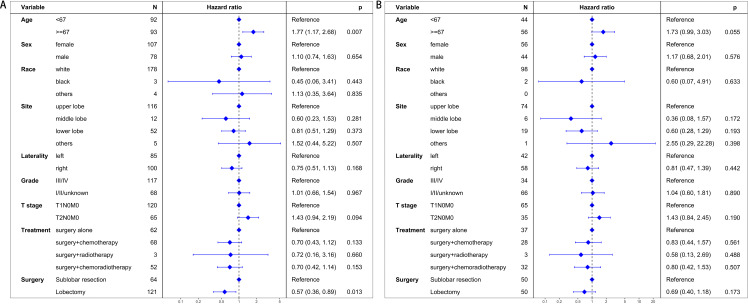
Multivariate regression analysis assessing prognostic factors for overall survival. (A) The unmatched cohort: This analysis identifies lobectomy as an independent predictor of improved overall survival in the unmatched cohort. (B) The propensity score matching cohort: This panel shows the adjusted analysis after matching, revealing that the survival benefit of lobectomy is not statistically significant once patient characteristics are balanced.

Before PSM, the median OS was 31 months for the sublobar resection group and 84 months for the lobectomy group. Univariate analysis indicated a significant OS benefit for lobectomy (HR = 0.58, 95% CI: 0.39–0.85, P = 0.005), as demonstrated in Fig 5A. This advantage was further supported by multivariate analysis, which identified lobectomy as an independent predictor of improved OS (HR = 0.57, 95% CI: 0.36–0.89, P = 0.013, Fig 6A).

Post-PSM, the median OS for the sublobar resection group was 30.5 months, compared to 60 months for the lobectomy group. Despite the apparent difference in survival times, the comparison between surgical approaches did not reach statistical significance (HR = 0.75, 95% CI: 0.46–1.21, P = 0.234), as shown in Fig 5B. This result was corroborated by multivariate analysis, which also failed to demonstrate a statistically significant difference in OS between the two surgical techniques (HR = 0.69, 95% CI: 0.40–1.18, P = 0.173, Fig 6B).

## Discussion

This retrospective cohort study demonstrates that, although sublobar resection was associated with inferior CSS and OS compared to lobectomy in patients with stage T1-2N0M0 SCLC, these differences did not reach statistical significance after PSM. This suggests that, while lobectomy may offer superior outcomes in some cases, the advantage may not be as pronounced once confounding factors are balanced.

Advances in diagnostic imaging, including thin-section computed tomography and 18-fluorodeoxyglucose positron emission tomography/computed tomography, have significantly enhanced the staging and early detection of non-small cell lung cancer. These technological improvements have led to better identification of early-stage lung cancers, allowing for more accurate treatment decisions. Historically, the landmark 1995 study by the Lung Cancer Study Group reported that sublobar resection was inferior to lobectomy in early-stage NSCLC, prompting lobectomy to become the standard of care for such cases, particularly for tumors larger than 2 cm [12].

In contrast, recent pivotal trials have challenged this view, particularly in small NSCLC lesions. The Japanese Clinical Oncology Group/West Japan Oncology Group (JCOG0802/WJOG4607L) and the Cancer and Leukemia Group B (CALGB140503) trials have provided robust evidence supporting the efficacy of sublobar resection for peripheral lung cancers measuring ≤ 2 cm [13,14]. In particular, the JCOG0802/WJOG4607L trial highlighted a survival benefit associated with segmentectomy, suggesting that preserving lung parenchyma could offer advantages, such as better postoperative pulmonary function and reduced morbidity [13]. The JCOG1211 trial further extended the potential role of sublobar resection to NSCLC up to 3 cm in size, especially when the tumor presents as a ground-glass opacity dominant lesion, which tends to be less aggressive [15].

Substantial benefits of sublobar resection, particularly for early-stage NSCLC, include the preservation of lung function, reduction in surgical morbidity and mortality, decreased intraoperative blood loss, and shorter hospital stays. These factors make sublobar resection an attractive option for select patients, particularly those with poor baseline lung function or other comorbidities that increase surgical risk.

However, SCLC is biologically distinct from NSCLC due to its rapid growth, high metastatic potential, and aggressive clinical course [16]. As a result, the findings from NSCLC studies may not be directly applicable to SCLC. In this study, sublobar resection was associated with poorer survival outcomes compared to lobectomy in patients with stage T1-2N0M0 SCLC. This suggests that the limited resection offered by sublobar surgery may be inadequate for the aggressive nature of SCLC, which often requires more comprehensive treatment approaches to achieve optimal outcomes.

Our analysis grouped all sublobar resections together, without distinguishing between segmentectomy and wedge resection, due to the small sample size. Among the 64 patients who underwent sublobar resection, 51 patients (79.7%) received wedge resection, while 13 patients (20.3%) underwent segmentectomy. Subgroup analysis revealed no statistically significant differences in CSS (P = 0.980) or OS (P = 0.654) between wedge resection and segmentectomy. The small number of segmentectomy cases severely limits the statistical power to detect meaningful differences between the two techniques. Merging them into a single group avoids overinterpreting underpowered subgroup results. Our study suggested that wedge resection and segmentectomy may yield comparable outcomes for early-stage SCLC. Merging them into a single group did not impact the results of our study.

Due to the limitation of SEER database, information of adjuvant chemotherapy or radiotherapy could not be extracted. To account for adjuvant therapy’s potential confounding effects, we included adjuvant chemotherapy and radiotherapy as covariates in our multivariable Cox regression models. The results demonstrated that adjuvant therapy was not an independent predictor of CSS or OS. This suggests that variations in adjuvant treatment intensity or regimens did not significantly influence the survival differences observed between lobectomy and sublobar resection.

Patient factors, such as comorbidities and performance status, may also influence the choice of surgical approach and subsequent outcomes. Cigarette smoking, the primary risk factor for lung cancer and a significant contributor to SCLC development, is associated with poorer lung function and increased comorbidities [17]. These factors can complicate surgical decision-making, leading to the selection of sublobar resection for patients deemed at higher surgical risk. However, the SEER database does not provide detailed information on patient comorbidities or smoking history, limiting our ability to fully explore these factors and their impact on survival outcomes [18]. Despite the use of PSM to minimize confounding, the lack of significant differences in CSS and OS between sublobar resection and lobectomy post-PSM should be interpreted cautiously. The rarity of surgically treatable SCLC limits the sample size, which affects the generalizability of our results.

In conclusion, while lobectomy demonstrated a trend toward superior survival in unmatched analyses, the lack of statistical significance after PSM underscores the need for individualized decision-making. Lobectomy may remain preferable for patients with adequate pulmonary reserve, whereas sublobar resection could be considered for high-risk individuals. Larger, prospective studies are warranted to validate these findings and refine patient selection criteria.

## Supporting information

S1 File Original data. (XLSX)
